# Cerebral organoids and their potential for studies of brain diseases in domestic animals

**DOI:** 10.1186/s13567-021-00931-z

**Published:** 2021-05-03

**Authors:** Bertrand Pain, Camille Baquerre, Muriel Coulpier

**Affiliations:** 1 Univ Lyon, Université Lyon 1, INSERM, INRAE, Stem Cell and Brain Research Institute, U1208, USC1361, Bron, France; 2 UMR1161 Virologie, Anses, INRAE, École Nationale Vétérinaire D’Alfort, Université Paris-Est, Maisons-Alfort, France

**Keywords:** Brain, Cerebral organoids, Domestic animals, Pluripotent stem cells, Neurotropic virus, Encephalitis

## Abstract

The brain is a complex organ and any model for studying it in its normal and pathological aspects becomes a tool of choice for neuroscientists. The mastering and dissemination of protocols allowing brain organoids development have paved the way for a whole range of new studies in the field of brain development, modeling of neurodegenerative or neurodevelopmental diseases, understanding tumors as well as infectious diseases that affect the brain. While studies are so far limited to the use of human cerebral organoids, there is a growing interest in having similar models in other species. This review presents what is currently developed in this field, with a particular focus on the potential of cerebral organoids for studying neuro-infectious diseases in human and domestic animals.

## Introduction: the brain, a complex organ

The brain is a complex organ and its access is delicate both during development and post-natal life of individuals, in all species. Reconstituting the development of this highly complex organ is a major challenge in neuroscience.

Embryonic development is a unique morphological process, with precise spatiotemporal control of the establishment of complex structures. In humans, the brain is the organ for which this process is the longest, slowest, and most complex. The neural tube is formed from a simple pseudostratified epithelial sheet, known as the neuroepithelium. It then extends and differentiates to give rise to the different parts of the central nervous system (CNS), including the anterior brain, which will generate, in mammals, the cortical hemispheres and striatum. The neural induction process begins on day 12 of gestation, with the establishment of the primitive line. Then, neurogenesis is initiated from week 8 of gestation and the first synaptic connections are established from weeks 11 to 12 [1]. Glial cells appear later: astrocytes and oligodendrocytes are detected from weeks 13 and 19, respectively, and the process of myelination does not begin until after birth. These observations illustrate the difficulty of recapitulating all the differentiation and maturation stages of this tissue which is particularly complex in its architecture and cellular interactions.

The initial mechanisms of formation of the neuroepithelium, neural tube, and cerebral vesicles are conserved in all vertebrates. They ultimately lead to the same basic architecture of the different parts of the brain (hindbrain, midbrain, and forebrain) in all vertebrates. The development of the forebrain is, however, more specific to mammals, although its volume and relative weight varies greatly between species. The significant enlargement of the frontal cortex is a specificity of primates, with an hyper-development in humans [2, 3].

Comparison of the brain structures of different mammalian species is usually performed between rodents, non-human primates (NHP) and humans. It is more rare with other large mammals although several magnetic resonance imaging (MRI) studies and brain atlas have been published for pig [4, 5], bovine [6], ovine [7, 8] and horse [9]. The encephalization quotient (EQ) and cerebellar quotient (CQ) parameters, defined as theoretical ratio between observed and expected total brain size (or cerebellum size for the CQ) at a given body mass [10] allowed to compare the size of mammalian brains in various species, including those of domestic animals [6, 11]. The similarities in the development of the human and pig brains is particularly interesting as it makes it possible to provide models that are more accessible than non-human primates (NHP). This interest has in fact been underlined by various authors “pigs have a large, gyrencephalic brain that can be studied using clinical MRI scanners/protocols. Pigs are less complex than non-human primates thus satisfying the “replacement” principle of animal research” [5] and also that “the large increase in brain volume in the postnatal period is similar to that of human neonates and suggests pigs can be used to investigate brain development” [12]. Of note, a recent study on NudE Neurodevelopment Protein 1 (NDE1) gene-whose protein is involved in dynein function and whose mutations are associated with human microhydrancephaly and lissencephaly-showed that a human-linked alternate terminal exon could be responsible for the appearance of gyri in mammals, including in pigs and bovine [13].

Some studies have focused on the brain of domestic animals (or on its sub-structures: hypothalamus, substantia nigra, sculum, cerebellum…), including bovine [6, 14], ovine [7, 15, 16], horse [9, 17] and pigs [18–21]. These studies are mainly focused on the description of innervation systems, distribution of specific neurotransmitters [22, 23] and functional connectivity [24]. Recently, the original report on the “restoration of vascular dilatory and glial inflammatory responses, spontaneous synaptic activity, and active cerebral metabolism in the absence of global electrocorticographic activity” in 4 h post-mortem pig brain emphasizes the importance of the pig model for innovative studies that explore new experimental and ethical ways of considering death [25].

At the molecular level, transcriptomic and proteomic analyses have also provided comparisons of gene and protein expression profiles between humans and different animal species [26, 27]. One study demonstrated that the composition of neurons and glial cells varies in pigs depending on the species [21]. Recently, the development of brain organoids from macaque and chimpanzee pluripotent stem cells (PSCs) have allowed the identification of certain aspects of the specificity of humans and great apes by comparative analysis at the single-cell level [28, 29]. The development and maturation of cortical structures is slower and more complex in the human model, although the gene activation and signaling pathways appeared generally well conserved between humans and non-human primates (NHP). For domestic animals, studies comparing the profiles of receptors for growth factors and cytokines in human, mouse and pig brains, such as performed by Sjöstedt et al. [26], can serve as a basis for the development of brain organoids in pigs. The precise identification of developmental signaling pathways will be a key element in obtaining the most structured and functional organoids, as it has been demonstrated in human.

## Cerebral organoid: an organ-like in vitro model that recapitulates key features of developmental brain

**Figure 1:**
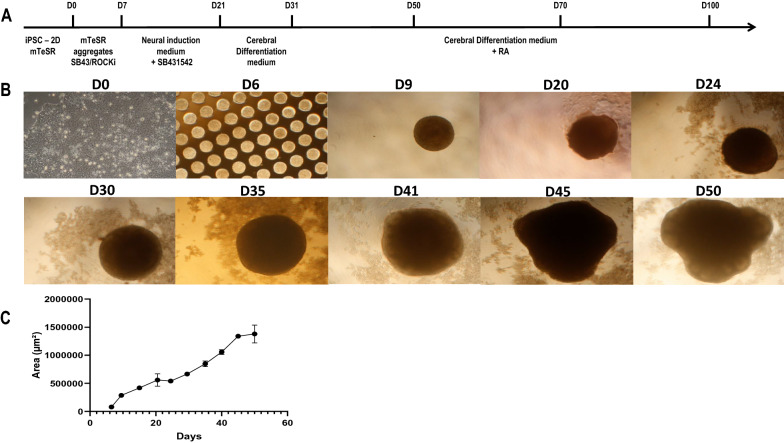
**Brain organoids obtained from in vitro differentiation of human-induced pluripotent stem cells (hiPSCs).** **A** Typical time course for obtaining cortical organoids. **B** Progressive growth of cortical organoid from hiPSC (D0, magnification ×100) to primary induction (D6–magnification ×100) and consecutive growth (D9-D50 magnification ×40). **C** Growth curve of cortical organoids (6 to 9 organoids per time point).

**Figure 2:**
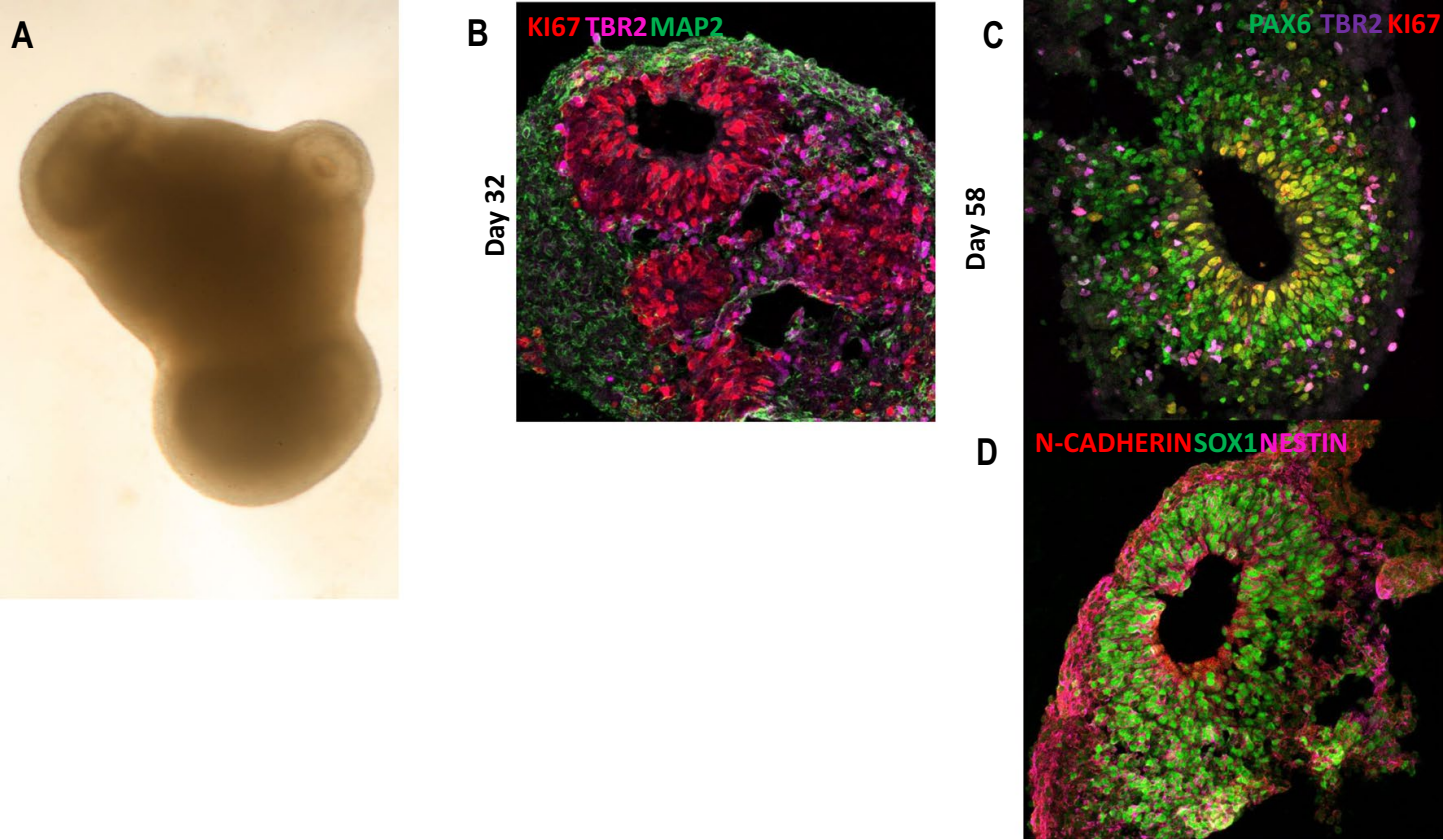
**Analysis of brain organoids.** **A** Typical cortical organoid obtained after 62 days of culture with apparent rosettes at the periphery (magnification ×100). **B**–**D** Immunofluorescence with antibodies directed against KI67, SOX1, NESTIN, TBR2, PAX6, MAP2 and N-CADHERIN, showing proliferative cells (KI67), progenitor cells (SOX1, NESTIN, TBR2, PAX6) and neurons (MAP2, N-CADHERIN) in **B** 32 days-old and **C**, **D** 58 days-old organoids.

Coming mainly from three areas of research, stem cells, cell culture engineering and developmental biology, organoids are defined as self-organized three-dimensional biological systems recapitulating the structure and cell types of the organs they aim to mimic as well as some of their functions. The most significant advances in the development of cerebral organoids were made with the pioneering work of Lancaster et al. in 2013 [30–32]. Their work was based on the following principle: human embryonic or induced pluripotent stem cells (iPSCs) are induced in the neural pathway in a controlled manner. Then the structure is allowed to self-organize and differentiate into a multilayered lamellar organoid similar to that observed during the development of the CNS. These first studies highlighted the need to follow a multistep protocol with various growing conditions. Aggregates were either maintained in the presence of Matrigel, a matrix that keeps the organoid in suspension and avoids polarization and attachment, or grown as free-floating structures. Suspending these drops in a stirred bioreactor allowed the maintenance of their development for several weeks. A diverse set of markers of proliferation and differentiation, including early progenitor markers such as SOX2, PAX6, TBR1, NESTIN and later expressed neural markers such as TUJ1, MAP2, CTIP2, SATB2, etc.., were used to verify the stage of differentiation of the growing organoids. More importantly, as the “human brain development exhibits a unique progenitor zone organization” i.e. the original presence of the outer subventricular zone (OSVZ) typical of the human and NHP neocortex [33, 34], it was concluded that the observed 3D structure of those cerebral organoids could recapitulate at least some aspects of the human early cortical development [30]. As patterning cues were identified, it was also possible to obtain cerebral organoids representative of different brain areas, including the forebrain, midbrain, and hypothalamus, described by Qian’s protocols [35]. Subsequently, several other approaches and techniques have allowed to reproduce more regions of the human brain [36]. All of these protocols follow similar steps with the first consisting of aggregated cells (formation of embryoid body (EB)-like structures), a second one with a primary induction into the neuroectoderm lineage, and a third one with the growth and amplification of the organoid followed by a phase of maturation that could vary from a few weeks to several months. Cerebral organoids may thus be generated either in a spontaneous (unguided) or a more controlled (guided) manner. This is illustrated in Figure 1, for cortical organoids as an example with the analysis of different markers during the maturation process (Figure 2). Various steps and changes in growth culture media, with specificity at each step, are shown. Growth factors and culture conditions are the main levers of action for controlling the specifications of the generated structures, with for example the absence of Vitamin A in the first steps and its presence for maturation ones (Figure 1). Addition of specific inhibitors of the WNT (IWR1e) and TGFβ (SB431242) pathways are often used for obtaining dorsally patterned forebrain organoids [37, 38], whereas inhibition of WNT (with IWP2) and activation of the Hedgehog pathway (with Activates Smoothened—SMO chemicals) allowed to obtain ventrally patterned forebrain organoid [39]. A non-exhaustive list, as synthetized and exemplified by Qian et al. [36], includes the unguided first cortical organoids [30, 31] and the same guided ones [30, 37], the cerebral cortex organoids [37], the various telencephalic structures [40], the cerebellum structures [41], the forebrain organoids [35, 42], the choroid plexus-like organoids [43], the hippocampus structure [35, 43], the midbrain organoid [44], the anterior pituitary organoid [45], the dorsal and ventral organoid [39, 46] and the recent choroid plexus- cerebrospinal fluid organoid (ChP-CSF) that mimics the CNS barrier [47]. The most recent advances have focused on assembloids which consist of either mixing/fusing dorsal and ventral forebrains [39] or mixing human medial ganglionic eminence (MGE) with cortical organoid, the latter one being used for modeling human interneuron migration [48].

Importantly, transcriptomic analyses have demonstrated that the various and numerous cell types found in the brain were also identified in the human 3D in vitro structures [49]. Acquisition of diversity was shown to be progressive and, over a period of 6 months of culture, most cell types were found to be related to the reference tissue. Single-cell RNA sequencing further revealed that human cerebral organoids recapitulated remarkably well the gene expression program activated during the development of the human fetal neocortex [38, 49, 50].

Another important feature of brain organoids is the demonstration of their functionality. For example, the typical interkinetic nuclear movement (a periodic movement of the cell nucleus in phase with cell-cycle progression which is observed in proliferative progenitors engaged in differentiation during cortical development) and electrical activities were observed in 3D cerebral organoids [51–53]. Oscillations were established and maintained beyond 25 weeks of culture and were detected by multi-electrode array recording. The authors reported that the electrical activity was similar to that recorded in a premature fetal brain [52].

Taken together, these recent reports established that numerous morphotypes present in the human brain are also found in the 3D structures and that standardized protocols allow reproducibility and robustness of the differentiation process in vitro, including in the long term [36, 47, 48].

## Biological issues addressed by cerebral organoids

Because of their organ-like features, human cerebral organoids are highly valuable in vitro models that are increasingly used for studies on neurodevelopment, disease modeling (e.g., neurodegenerative diseases, infectious diseases, cancer), toxicity and drug testing [54, 55]. Compared to 2D models, they have several advantages. The cell-to-cell communication and extracellular matrix, which are essential to respond to the environment and to transfer information from one cell to another and which play an important role in physiological processes, are more faithfully modeled. They also present a high diversity in neural population that is not encountered in 2D models. Today, they are the closest in vitro model of the human brain. They have assisted in the understanding of several neurological diseases: those of embryonic origin such as autism [42, 56, 57], genetic or viral-induced microencephaly [30, 35, 58], lissencephaly and Miller-Dieker syndrome [59, 60], but also those of the aging brain such as Parkinson’s disease [61–63], Alzheimer’s disease [64], stroke [65, 66] and brain tumors such as glioblastomas [67–69]. They are also expected to be more predictive for drug development. Indeed, while 2D models are easier to handle and have been largely used for drug discovery, they have also been shown to be of low predictive value, as they led to a high level of failure in clinical assays.

### Human cerebral organoids for the study of neurotropic infectious diseases

Another application for human brain organoids that is currently expanding and which may be translated to the veterinary sciences concerns the study of infectious diseases and host–pathogen interactions [70]. Cerebral organoids showed their high potential when the Zika virus emerged in South America in 2016 and was declared a public health emergency by the World Health Organization because of its possible association with multiple cases of microcephaly in newborns [71]. Studies using cerebral organoids have been used to demonstrate the causal role of the Zika virus in this devastating disease and to elucidate the mechanisms that lead to neurogenesis impairment [35, 72]. Brain organoids have also been used to test drugs to prevent or treat infection [73]. These works have paved the way for studies of other viruses that are involved in neurodevelopmental diseases, such as the human cytomegalovirus (hCMV), and possibly the Borna disease virus for which an alteration in neurogenesis was shown in a 2D in vitro model [74, 75]. Human CMV infection has recently been modeled using brain organoids and developmental alterations, that are comparable to those observed in hCMV clinical samples, have been described, demonstrating the utility of this model for further investigation [76]. Studies of neurotropic viruses are not restricted to those impairing neurodevelopment. They can be extended to viruses, or more generally to pathogens or infectious proteins, that are responsible for encephalitis or other neurological diseases. Indeed, the cellular heterogeneity of the 3D structures, which comprise different types of neurons, including dopaminergic, glutamatergic, and GABAergic neurons, as well as neural progenitor and glial cells, allows studies that aim at understanding neuropathogenic mechanisms in the more mature brain. Such studies were performed with the Japanese encephalitis virus (JEV), an encephalitic flavivirus transmitted by mosquitoes which is responsible for more than 70 000 human cases each year with more than 10 000 fatalities. It is so far poorly understood why the outcome of JEV infection is more severe in children than in adults, but answers may come from modeling the infection with brain organoids. A first study has indeed given some clues, revealing a preferential tropism for radial glia and astrocytes and a weaker antiviral response in the youngest organoids compared to older one [77]. Another example is given by the recent modeling of the herpes simplex virus 1 (HSV1) infection in brain organoids, allowing studies of the virus trafficking through the complex neural tissue structure, the processes of latency and reactivation that can be established in organoids [78, 79] and the role of the virus as a facilitating agent of Alzheimer disease. Interestingly, HSV1 infection in both 2D and 3D in vitro models derived from iPSCs showed that infection led to the deposit of amyloid β42 aggregates, supporting the hypothesis that HSV1 may be involved in the occurrence of Alzheimer disease [78–80]. However, in 2D cells, aggregates were observed in infected cells whereas they were present only in non-infected cells in the 3D model. This showed differences that have strong implication for understanding the neuropathology of this infection, leading the authors to consider that the 3D model may be more relevant for studies aiming at understanding the role of HSV1 as a facilitating factor of Alzheimer disease.

Brain organoids may also be used to determine whether a virus has neurotropic properties. This is a question that has been raised for the novel severe acute respiratory syndrome coronavirus 2, responsible for the current coronavirus disease pandemic [81–84]. This question appears to be of dramatic importance as understanding whether the neurological symptoms observed in some of the infected patients are due to inflammatory process or by the virus entering the cerebral parenchyma will determine the most appropriate therapy. Viruses are not the only neurotropic pathogens which can take advantage of cerebral organoids. Great advances in the study of human prion diseases is also expected in the future thanks to their use. Modeling human prion propagation and disease has indeed been highly challenging so far as cell lines were not capable of generating PrPres, the infectious form of the cellular prion protein (PrPc). Interestingly, not only the generation of organoids using iPSCs derived from patients carrying a mutation in the PrPc gene, which predisposed them to prion disease, allowed the modeling of certain pathological changes [85, 86], but modeling the generation and propagation of infectious prion was achieved using 5-months old cerebral organoids [85]. As diverse brain regions with specific neuronal population (dorsal and ventral forebrain, cerebellum, etc.…) can be generated using corresponding patterning cues [36, 39, 46], it will now be possible to question the mechanisms by which prion strains specifically target certain neuronal population, a question that is so far unresolved and which can be extended to numerous viruses which, like prions strains, infect specific neuronal population. So far, cerebral organoids were used for modeling only a few of the hundred viruses and other infectious agents that are capable of invading the human brain and for which a better understanding of their pathogenic actions is necessary to develop therapeutic drugs that will prevent or limit the irreversible damage they induce in the brain. One can extrapolate that, in the near future, more virologists will collaborate with neuroscientists who have developed brain organoids and will benefit from this highly innovative tool to both improve our understanding of brain–virus interactions and to test antiviral and neuroprotective drugs.

### What is the situation in domestic animals?

Cerebral organoids are so far restricted to primate and rodent species. As neurological diseases and neurotropic infection, affect domestic animals and since data obtained with one species are not fully transposable to another one, cerebral organoids specific to domestic animals will be useful for veterinary research. They may also be highly informative for developmental biology. Developing them will request robust and plastic PSCs. These cells have already been generated from many domestic species [87, 88], including chickens [89–91], rabbits [92, 93], sheep and cattle [94–96], pigs [97–100], dogs [101], and horses [102, 103], however, they sometimes appear less robust than murine and human PSCs. Nevertheless, progress has been made and reports with recently developed PSCs from horses [103], dogs [101], pigs [100] and bovine [96] show an improved plasticity and therefore open new perspectives. More details on their isolation and properties will not be given here as they are already presented and discussed in the introductive chapter of this issue [104].

Below, we will give an overview of the zoonotic and non-zoonotic neurotropic viral infection which affect domestic animals and for which studies using cerebral organoids may be useful. Next, we will give few examples of specific questions that could be tackled using them.

Neurotropic viruses affecting domestic animals belong to many families and genera, including *Lyssavirus*, *Flavivirus*, *Alphavirus*, *Bunyavirus*, etc. They are summarized in Table 1. The rabies virus, which belongs to the *Lyssavirus* genus, is probably the most well-known of the neurotropic viruses. It is capable of infecting all mammals, but dogs, wild carnivores, and bats are considered its natural reservoirs. Notably, bats are the natural reservoir of at least 12 of the 14 identified species of *Lyssavirus* [105]. In 99% of cases, the transmission to humans occurs through dogs. It induces an acute and progressive encephalitis, with almost 100% lethality [106]. The vesicular stomatitis virus is another *lyssavirus* that affects pigs, cattle, and horses in the Western hemisphere. It is rarely zoonotic but infection in children has been described [107]. The *Flaviviruses* of the *Flaviviridae* family form another genus that comprises many neurotropic viruses. They induce encephalitis in humans and horses (West Nile virus, Japanese encephalitis virus, Saint-Louis encephalitis virus, Murray Valley encephalitis virus…) or sheep (Louping Ill virus) [108]. Louping Ill-induced encephalitis are however rare in humans [109]. Alphaviruses and bornaviruses also comprise neurotropic viruses that can cause serious diseases in both humans and horses [110]. The former genus includes the Eastern, Western, and Venezuelan equine encephalitis viruses, and the latter includes the mammalian 1 orthobornavirus [111]. The henipaviruses, whose emergence occurred in the 1990s, also cause encephalitis in humans and animals with very high fatality rates in both of them. The Nipah virus and Hendra virus target pigs and horses, respectively. Humans are infected either directly from bats (their natural reservoir) or by contact with infected pigs and horses (their amplifying host) [112, 113]. Among the non-zoonotic viruses, the Schmallenberg virus, a *Bunyavirus* transmitted by *Culicoides* vectors, appeared in Germany in 2001, affecting ruminants (mainly bovine, but also sheep, goats, and wild ruminants). It induces neurological damage such as blindness, ataxia, paralysis, and sometimes convulsions and can also cause severe deformations of the calf brain during development [114]. The equine herpes virus 1 is distributed worldwide and is responsible for myeloencephalopathy in horses [115] and the porcine hemagglutinating encephalomyelitis virus is a coronavirus that induces encephalomyelitis in piglets under 3 weeks old [116].

**Table 1:** **Main zoonotic or non-zoonotic neurotropic viruses affecting domestic animals**

Virus family/Genus	Virus	Domestic species affected, (Z)	Confirmed/suspected reservoir	Reference
*Rhabdoviridae*				
* Lyssavirus*	Rabies virus (RABV)	Mammals, (Z)	Bat	[106]
* Lyssavirus*	Vesicular stomatitis virus (VSV)	Pig, cattle, horses (Z)		[107]
*Flaviviridae*				
* Flavivirus*	Japanese encephalitis virus (JEV)	Pig, horses, chicken, (Z)	Mosquitoes	[108]
	West Nile virus (WNV)	Pig, cattle, sheep, horse, dog, cats (Z)	Mosquitoes, Corvidae	[108]
	Louping Ill virus (LIV)	Sheep, (Z)	Ticks	[108, 109]
	Tick-borne encephalitis virus (TBEV)	Cattle, goats, Horses, (Z)	Ticks, small rodents	[108]
*Alphaviridae*				
* Togavirus*	Eastern equine encephalitis virus (EEEV)	Horses, (Z)	Mosquitoes	[108]
	Western equine encephalitis virus (WEEV)	Horses, (Z)	Mosquitoes	[108]
	Venezuelan equine encephalitis virus (VEEV)	Horses, (Z)	Mosquitoes	[108]
*Bornaviridae*				
* Orthobornavirus*	Borna disease virus 1 (BoDV-1)	Horses, sheep, squirrels, (Z)	Bicolored white-toothed shrew	[108]
*Paramixoviridae*				
* Henipavirus*	Nipah virus (NiV)	Pigs, (Z)	Bats	[113]
* Henipavirus*	Hendra virus (HeV)	Horses, (Z)	Bats	[113]
*Buynaviridae*				
* Bunyavirus*	Schmallenberg virus (SBV)	Cattle, Sheep, goat	Culicoides	[114]
*Herpesviridae*				
* Alphaherpesviridae*	Herpes virus équin 1 (HVE 1)	Horses		[115]

The main economic losses induced by viruses in domestic animals are not due to neurological disorders with the noticeable exception of horses which are strongly affected by numerous neurotropic viruses, including Eastern, Western and Venezuelan equine encephalitis viruses in North and South America [117].

Among all species affected by neurotropic infection, horses may be the most appropriate for the development of cerebral organoid. First, because the capacity to differentiate into the neural lineage and to generate neurons have already been shown for equine iPSCs [118]. Second, because horses drive economical and affective issues that attract the funding necessary for this research and third, because numerous viruses affect the equine brain [108]. In addition, horses suffer from neurological diseases of non-infectious etiologies [119], which will also benefit from this novel in vitro approach. Similar research developed in human organoids, aimed at understanding key cellular and molecular drivers of the pathobiology and at developing therapeutic drugs is expected. Importantly, as many viruses targeting the equine brain are zoonotic, research on human and equine cerebral organoids infected by the same viruses will drive knowledge on cellular signaling pathways that are either similar or on the contrary specific to each species. This may lead to the development of drugs usable in both species. Development of ovine cerebral organoids is also highly attractive, as this will allow studies aiming at understanding the mechanisms of propagation of atypical scrapie for which there is, so far, no in vitro model available. In particular, it may help to understand why the ARR/ARR ovine genotype, which is highly resistant to classical scrapie is, on the contrary, sensitive to atypical scrapie. As cerebral organoids of human origin have shown their strong potential for propagating infectious prions known to be highly difficult to propagate in other in vitro models, it is expected that propagation of ovine prion will also be possible. This will bring new knowledge on ovine prion diseases that are under strong genetic control of the prion PRNP gene. Pet domestic animals will also greatly benefit from the use of cerebral organoid. Dogs for example, are subjected to brain diseases such as cognitive deficits that are similar to Alzheimer disease in humans [120] and brain tumors [121], for which there is currently no cure. Modeling these diseases in canine cerebral organoids, as it was performed for similar human diseases, is expected to help understanding the species-specificities and to test and develop new therapeutics.

These are a few examples of many questions that can be tackled using cerebral organoids from domestic animals. Veterinary research will thus take advantage of this new technology to address questions that are specific to animal diseases. Importantly, this will also allow to diminish the use of animals, complying with the 3Rs. The research costs will also considerably decrease as maintaining large animals for long periods, a necessity for brain diseases studies, is highly expensive compared to in vitro cultures. Finally, the proximity of certain animal brain diseases with human diseases may allow veterinary research to benefit human research, and conversely, in a One Health spirit.

## Future and limits of cerebral organoids

The complexity and processes of brain development have been extensively studied in humans, NHPs and ferrets [122] but little is known in domestic animals. However, as we mentioned it previously, comparative studies have demonstrated a strong proximity of brain structures in different mammal species, suggesting that similar cellular and molecular mechanisms underlie cerebral development in mammals. It can thus be hypothesized that protocols developed to obtain human brain organoids could be transposed to domestic species. Such transposition was already shown to be successful for pigs, rabbits and bovine intestinal organoids [123]. As also mentioned, PSCs have been generated from many animal species [87, 88, 104]. The pluripotency and differentiation properties of some of the reprogrammed cells in these species are, however, not similar to those of mouse and human models. In other words, many of these cells are imperfectly reprogrammed and cannot fully engage in the differentiation pathways, with the exception of those reported in horses (for which accurate differentiation and infection by Flaviviruses have been reported) [118], in dogs [101] and those recently isolated in pigs [100] and bovine [96]. One can hope that, in the future, the development of more reprogrammed cells with improved plastic properties of differentiation will be available to generate brain organoids in the different species of interest. This would allow to question the neuropathogenic mechanisms of viruses in domestic animal models that are physiologically relevant and would be highly beneficial for veterinary research.

Recently developed in humans, cerebral organoids constitute a powerful and exciting tool that can be used for various applications from disease modeling to drug testing. The latest developments, such as the constitution of assembloids (brain structures generated independently and interconnected), which allow to follow the neural dynamics of the interactions between different brain areas [39, 124–126]), mimicking the in vivo interconnections [48, 127] will increase their value further. However, cerebral organoids also have limitations. The brain is associated closely with the vascular system and mimicking the blood–brain barrier remains a challenge that needs to be resolved [47, 128, 129]. The first organoid vascularization assays during transplant experiments in rodent brains are underway and demonstrate the possibility of integrating these structures with preexisting organs [130]. However, despite the rapid and impressive progress, the complex and complete physiology of the brain is still imperfectly mimicked, in particular with the absence of microglia, in the models developed currently [131].

Finally, in humans, the ethical dimension of cerebral organoids should be considered, especially with regards to the origin of cells, the consent of patients, and to the new ways of screening and identifying associated disorders that these new structures can produce. The notion of consciousness must also be posed for human organoids, as electrical activity similar to that observed in second-semester fetuses has been shown and as more mature wiring may be developed with the generation of assembloids [132–134]. For animals, the ethical dimension is clearly different since consent of patients for obtaining the cells to be reprogrammed is not an issue. Importantly, the accessibility to organoids will undoubtedly leads to a reduction in the use of animals in experimental protocols, satisfying the 3R rule. This will certainly be a major driven force for conducting researches aiming at improving animals PSCs and developing organoids.

## Conclusion

The availability of human PSCs (ESCs and iPSCs) and the development of protocols that allow the generation of 3D structures with some of the brain’s functionalities have led to human cerebral organoids which became an indispensable tool in neuroscience. Veterinary research is however not as advanced. Despite the generation of iPSCs in different species, their weak quality and lack of plasticity has so far been insufficient for the development of cerebral organoids. The recent progress in the generation of new PSCs with improved differentiation potential has however raised hope for their effective development in domestic animal species. Drug discovery for animal brain diseases, especially infectious diseases, will greatly benefit from this innovation.

## Abbreviations

CNS: Central nervous system
ESCs: Embryonic stem cells
iPSCs: Induced pluripotent stem cells
PSCs: Pluripotent stem cells
